# 877. Synovial Fluid Testing Using the BioFire® Joint Infection (JI) Panel Potentially Decreases Time to Antibiotic Optimization for Acute Joint Infections

**DOI:** 10.1093/ofid/ofac492.070

**Published:** 2022-12-15

**Authors:** Benjamin Whitt, Abdullah Kilic, Elizabeth Palavecino, James Beardsley, Christopher Ohl, Tyler Stone, Alex D Taylor, Vera Luther

**Affiliations:** Atrium Health Wake Forest Baptist, Winston Salem, North Carolina; Wake Forest University, winston salem, North Carolina; Wake Forest School of Medicine, Winston Salem, North Carolina; Atrium Health Wake Forest Baptist, Winston Salem, North Carolina; Wake Forest School of Medicine, Winston Salem, North Carolina; Atrium Health Wake Forest Baptist, Winston Salem, North Carolina; Atrium Health Wake Forest Baptist, Winston Salem, North Carolina; Wake Forest School of Medicine, Winston Salem, North Carolina

## Abstract

**Background:**

Traditional synovial fluid culture for acute joint infection (AJI) is time-consuming, potentially delaying initiation of targeted antimicrobial therapy. The BioFire® Joint Infection (JI) panel, a multiplex PCR for synovial fluid, has been shown to have 90.2% sensitivity and 99.8% specificity for diagnosis of AJI. We sought to determine the potential impact of the JI panel on antimicrobial use for AJI at our institution.

**Methods:**

Antimicrobial use was retrospectively reviewed in a cohort of adult inpatients who underwent synovial fluid culture and were diagnosed with septic arthritis or periprosthetic joint infection from Sept 2021-Apr 2022. Patients were excluded if there was an alternative source of infection or septic shock. Synovial fluid specimens were tested using the JI panel, and results were compared to those of traditional culture methods. Patients who were initially treated with broad-spectrum antimicrobials for AJI were included in a review of antimicrobial prescribing. Records were reviewed to determine the time at which a transition to targeted therapy was made when using culture. This was compared to the time at which panel results could be available and acted upon, in this study presumed to be 12 hours after culture collection.

**Results:**

42 inpatients met study criteria for comparison of culture and JI panel results. The panel identified the organism that was grown in culture in 21 cases while detecting an organism in 5/16 culture-negative cases (table 1). In 5 cases, culture identified an organism not included on the JI panel. 29 patients met criteria for review of antimicrobial prescribing. Of these, therapy in 14/16 with positive cultures could have been changed earlier (table 2), resulting in an average reduction of time to targeted therapy of 57 hours (range 27–107 h, median 50.5 h) per patient. Targeted therapy could have occurred in 5/13 culture-negative cases.

Summary of comparison of culture and BJI panel results.

*Organisms identified by culture: Coagulase-negative staphylococci (n=4), Pasteurella multocida (n=1)

**Organisms detected by JI panel: Streptococcus pyogenes (n=2), Streptococcus agalactiae (n=1), Finegoldia magna (n=1), Enterobacter cloacae (n=1)

Proportion of patients eligible for a change in antimicrobial therapy.

**Conclusion:**

Use of the JI panel could have prompted earlier optimized therapy in approximately two-thirds of patients. In 5 instances where cultures were negative, important AJI pathogens were identified. Another potential benefit includes reduced exposure to unnecessary broad-spectrum antimicrobials. Future study should evaluate the actual impact of the JI panel in practice.

**Disclosures:**

**All Authors**: No reported disclosures.

